# Ethical Dilemmas Among Oncology Nurses in China: Cross-Sectional Study

**DOI:** 10.2196/63006

**Published:** 2024-12-13

**Authors:** Eunjeong Ko, Neda Shamsalizadeh, Jaehoon Lee, Ping Ni

**Affiliations:** 1 School of Social Work, San Diego State University, San Diego, CA, United States; 2 School of Nursing, San Diego State University (Imperial Valley Campus), San Diego, CA, United States; 3 Department of Educational Psychology, Leadership, & Counseling, Texas Tech University, Lubbock, TX, United States; 4 School of Nursing, Tongji Medical College, Huazhong University of Science and Technology, Number 13, Hangkong Road, Qiaokou District, Wuhan, 430030, China, 86 13871540316, 86 02783692635

**Keywords:** prognosis-related communication, ethical dilemmas, oncology, ethics, China, beliefs, nursing, cultural values, prognosis

## Abstract

**Background:**

Effective communication about cancer prognosis is imperative for enhancing the quality of end-of-life care and improving patient well-being. This practice is sensitive and is heavily influenced by cultural values, beliefs, and norms, which can lead to ethical dilemmas. Despite their significance, ethical challenges in nursing related to prognosis communication are understudied in China.

**Objective:**

This study aimed to examine the ethical dilemmas relating to cancer prognosis communication and their associated factors.

**Methods:**

A cross-sectional design was employed to survey 373 oncology nurses in mainland China. Data were collected on ethical dilemmas, attitudes, barriers, experiences with prognosis communication, sociodemographics, and practice-related information. Ordinary least squares regressions were used to identify factors contributing to ethical dilemmas.

**Results:**

Participants reported a moderate level of ethical dilemmas in prognostic communication (mean 13.5, SD 3.42; range 5‐20). Significant predictors of these dilemmas included perceived barriers (*P*<.001), experiences with prognosis communication (*P*<.001), and years of work experience (*P*=.002). Nurses who perceived greater communication barriers, had more negative experiences with prognosis communication, and had less work experience were more likely to encounter ethical dilemmas in prognosis-related communication.

**Conclusions:**

Chinese oncology nurses frequently encounter ethical dilemmas, as well as barriers, in communicating cancer prognoses. This study’s findings emphasize the importance of culturally tailored communication training. Collaborative interprofessional training, particularly through physician-nurse partnerships, can perhaps enhance the proficiency of cancer prognosis-related communication.

## Introduction

### Background

Prognosis-related communication, also known as truth-telling or breaking bad news, is a continuous process that encompasses discussing life expectancy, symptom progression, and functional abilities with patients, their families, and health care professionals [1]. Effective discussion about a prognosis facilitates informed decisions for patients and enhances patient-reported outcomes [2]. Despite the expectation that prognosis communication should be a standard practice and a universal communication ideal in health care, health care professionals, including nurses, experience discomfort and concerns in breaking bad news. Health care professionals are concerned about providing prognostic information that could contribute to emotional distress in patients and families. This raises questions about their professional roles in alleviating suffering and concerns about the potential impact on relationships [3].

Insufficient training, unclear nursing roles, inadequate communication skills, time constraints, and concerns about diminishing the patient’s hope [4-6] can impact prognosis communication. Interpersonal factors such as the patient’s and family’s lack of support, the family’s request to withhold information from their loved one, and lack of communication from physicians can influence this process [7,8]. Nurses often face ethical dilemmas when there is a disparity between their professional duties and the complex circumstances surrounding family beliefs and cultural norms, making it extremely difficult to proceed [7,9]. Such conflicts can yield negative consequences, including increased burdens, moral distress, emotional burnout, anxiety, and guilt [7,10].

Practices in prognosis disclosure vary by country and cultural groups, influenced by unique cultural beliefs and values [11-13]. In Western cultures, informing patients about their cancer diagnosis and prognosis is deemed vital for promoting patient autonomy and is routinely integrated into palliative care. However, in Eastern countries like China, individual autonomy is often considered secondary to family-centered decision-making [11,14]. Cultural values that prioritize family-centeredness lead health care professionals to defer disclosure of the prognosis to the family, who then decide whether to inform the patient [15]. It is common for families to withhold prognostic information to protect patients from emotional distress [15]. In addition, death and dying are taboo subjects, making it challenging for health care professionals to facilitate timely and effective prognosis-related communication due to concerns about potential adverse outcomes such as emotional distress, diminished hope, and acceleration of the dying process [12,16].

In countries where honest disclosure is not well recognized, health care professionals’ challenges and conflicts are greater. Chinese nurses often experience a conflict between personal dimensions influenced by traditional cultural norms versus professional dimensions of adhering to nursing values and principles [12]. Withholding end-of-life (EOL) communication from patients and relying solely on family members for EOL decision-making raises concerns, as families may not fully understand the patient’s prognosis or their EOL wishes [15,17]. Recent studies indicate that the majority of cancer patients in China want to be informed of their prognosis [8,18,19]. For example, a meta-analysis of studies in China revealed that 81.8% of patients with cancer, compared to 32.4% of the family, prefer being informed of the prognosis, but only 19.9% of patients are actually informed [8,15]. Although health care professionals aim to maintain hope through nondisclosure, they experience conflicts in balancing hope preservation with truth-telling [16]. The disparities between the professional duty to uphold patient autonomy and the cultural and traditional norms that define what is beneficial for patients give rise to ethical dilemmas and moral distress among health care professionals [17,20].

### Aim of the Study

In China, there were 4,546,400 new cancer cases and 2,992,600 cancer deaths in 2020, accounting for 25.1% and 30.2% of global incidences, respectively [21]. Despite the increasing rates of cancer diagnosis and mortality and the significant implications for nursing, there is limited knowledge about the ethical dilemmas and other factors related to prognostic communication among oncology nurses in China. Although several studies in China have examined the complexities of prognosis communication within a sociocultural context, most focus on the nurses’ attitudes and preferences for diagnosis or prognostic disclosure [22-24]. Addressing ethical dilemmas in nursing practice is imperative for upholding patient advocacy, promoting patient-centered care, navigating complex moral issues, and maintaining professional integrity and accountability. This study aims to explore the ethical dilemmas in prognosis-related communication among Chinese oncology nurses and to identify their influencing factors.

## Methods

### Study Design and Sample

This study employed a cross-sectional design using a web-based survey conducted with oncology nurses. Convenience sampling was used to recruit participants from 4 hospitals in Wuhan City, Hubei Province, mainland China. The 4 hospitals were tertiary hospitals, which are designated based on a 3-tiered system (primary, secondary, and tertiary). A tertiary hospital has more than 500 beds, offers specialized health care services, and plays a significant role in medical education and research. The inclusion criteria were registered nurses currently practicing in oncology units and working with patients with advanced cancer. The exclusion criteria included nurses with less than 1 year of practice in oncology, intern nurses, rotating nurses, and those working in pediatric oncology.

### Data Collection and Procedure

Our research team contacted nursing directors at 4 hospitals with oncology units in Wuhan and explained the purpose and procedures of the study. The nursing directors and the head nurse distributed the web-based survey to the oncology department’s WeChat (Tencent) group, inviting all oncology nurses to participate. The survey was administered from September 17, 2018, to October 24, 2019. A total of 410 eligible nurses enrolled in the study, but 37 did not fully complete the survey, resulting in a final sample of 373 participants.

### Ethical Considerations

This study was approved by the Ethics Committee of Tongji Medical College, Huazhong University of Science and Technology, IEC(S198), and all procedures followed ethics standards. Before taking the survey, nurses were informed about the study’s purpose, risks and benefits, and their right to withdraw, and then they provided consent to participate the study. Surveys were anonymous and the data were kept confidential. No compensation was given to the study participants.

### Measures

#### Overview of Measures

We developed measures by adapting items from a questionnaire used in previous studies [25,26]. The questionnaire was translated from English to Chinese and back translated to English by different postdoctoral staff independently to avoid bias. Some wordings and phrases were modified to enhance clarity in translation. Discrepancies were resolved with the help of a bilingual nursing faculty member. The content of the survey was validated by an expert panel consisting of 2 administrators with nursing backgrounds, 2 head nurses from an oncology department, and 2 oncologists with extensive knowledge and clinical experience in the study topic. These experts evaluated the clarity and relevance of the survey’s components for the study population and verified the translation of the survey. Their feedback was integrated to refine the items and translation. The translated questionnaire was pilot-tested with 25 oncology nurses to make further improvements. Item responses were summed to calculate 4 composite (scale) scores: dilemmas, communication experiences, attitudes, and barriers.

#### Dilemmas in Prognosis-Related Communication

This scale (4 items) assessed experiences with dilemmas in prognosis-related communication such as discomfort with discussions and social and cultural conflicts. Responses were rated on a 5-point Likert scale (1=never/almost never; 5=always/almost always). The total scores for ethical dilemmas ranged from 4 to 20, with higher scores indicating more dilemmas. The Cronbach α for this scale was 0.82.

#### Prognosis-Related Communication Experiences

This scale (3 items) measured experiences involving (1) patients not wanting their family members to be informed of their diagnosis, (2) family members or relatives requesting that patients not be told bad news, and (3) nurses not being encouraged to participate in prognosis-related communication. Responses were rated on a 5-point scale (1=never; 5=always/almost always). The total scores ranged from 3 to 15, with higher scores indicating more negative experiences in prognosis-related communication. The Cronbach α for this scale was 0.70.

#### Attitudes Toward Prognosis-Related Communication

This scale (6 items) measured attitudes toward engaging in prognosis communication using a 4-point scale (1=strongly disagree; 4=strongly agree). Sample items were “patients can make timely decisions about their treatment if they understand their prognosis” and “answering questions about prognosis-related information is within the scope of nursing practice.” The total scores ranged from 6 to 24, with higher scores indicating more positive attitudes. The Cronbach α for attitudes was 0.87.

#### Barriers to Prognosis-Related Communication

This scale (4 items) assessed perceived barriers to engage in prognosis communication, such as role uncertainty, lack of time, and fear of diminishing hope. Responses were rated on a 4-point scale (1=strongly disagree; 4=strongly agree), with higher scores indicating greater barriers. The total scores ranged from 4 to 16. Higher scores indicate greater levels of barriers in communicating prognosis. The Cronbach α for barriers was 0.80.

Prognosis-related practice questions included: (1) preference for disclosing prognosis (yes/no), (2) the person responsible for disclosing prognosis (physician, family, nurse, or other), and (3) the extent of previous prognostic information disclosure to patients and family (fully, partially, or avoid of disclosure). Lastly, sociodemographic and practice-related questions included age, sex, marital status, education level, years of oncology nursing experience, and formal training on prognosis communication (1=none/almost none; 4=a lot).

### Data Analysis

Descriptive statistics were used to demonstrate the distributions of participants’ sociodemographic characteristics and other study variables. For continuous variables, mean and SD were calculated, while for categorical variables, the count and frequency were reported. To identify factors associated with increased or decreased dilemmas when engaging in prognosis communication (outcome variable), an ordinary least squares regression was performed. Predictors included attitudes, barriers, and experiences in prognosis-related communication. This analysis controlled for several covariates, including participants’ age, sex, marital status, and education level to ensure an unbiased assessment of each predictor’s independent impact on the dependent variable—dilemmas in prognosis communication. All analyses were conducted using SPSS 23.0 (IBM Corp), and statistical significance was determined at an α level of .05.

## Results

### Sociodemographic and Practice-Related Characteristics

Table 1 presents the sociodemographic information about the study sample. The median age of the participants was 30 (IQR 28‐33) years. The majority were female (352/373, 94.4%), married (274/373, 73.5%), and had a bachelor’s degree (219/373, 58.7%). The median years of working as an oncology nurse was 5 (IQR 3‐8) years, with 65.6% (238/373) reporting that they had received little or no formal training in prognosis communication.

**Table 1.** Participant demographic and practice-related data.

Characteristics	Participants (n=373)
Age (years), median (IQR)	30 (28‐33)
Sex, n (%)	
Female	352 (94.4)
Male	21 (5.6)
Education, n (%)	
Secondary specialized school of nursing	50 (13.4)
Junior college nursing degree	97 (26)
Bachelor’s degree	219 (58.7)
Master’s/PhD^a^ degree	7 (1.9)
Marital status, n (%)	
Married	274 (73.5)
Separated/divorced	19 (5.1)
Widowed	4 (1.1)
Never married	76 (20.4)
Years working as an oncology nurse, median (IQR)	5 (3‐8)
Formal training for prognosis-related communication, n (%)^b^	
None/almost none	77 (21.2)
Little bit	161 (44.4)
Moderate	90 (24.8)
A lot	35 (9.6)

a PhD: doctoral degree in nursing.

b n (%) is based on 363 respondents due to missing values.

Regarding prognosis-related practice, 51.1% (228/373) of the participants believed that physicians should be responsible for delivering prognostic information, compared to 10.2% (38/373) who believed nurses should take on this role (see Table 2). Approximately 86.9% (324/373) of the participants engaged in prognosis communication, providing either full or partial disclosure to patients or their families. Only 20.1% (75/373) of the participants reported providing full disclosure to patients, 50.1% (187/373) provided partial disclosure, and 29.8% (111/373) avoided disclosure. In contrast, 43.7% (163/373) of the participants reported providing full disclosure to patients’ families, while 37.3% (139/373) provided partial disclosure.

**Table 2.** Prognosis-related practice.

Variables	Participants (n=373)
When patients have a poor prognosis, should this be disclosed to the patient? n (%)	
Yes	254 (68.1)
No	119 (31.9)
Who should inform about the prognosis? n (%)	
Physician in charge	228 (51.1)
Family member	90 (24.1)
Nurse in charge	38 (10.2)
Other	17 (4.6)
Prognosis communication to patients, n (%)	
Fully informed patients	75 (20.1)
Provided only partial information	187 (50.1)
Avoided informing prognosis/never disclosed prognosis to patients	111 (29.8)
Prognosis communication to families, n (%)	
Fully informed families	163 (43.7)
Provided only partial information	139 (37.3)
Avoided informing prognosis/never discussed prognosis to the families	71 (19)

### Ethical Dilemmas, Experiences, Attitudes, and Barriers Toward Prognosis-Related Communication

Table 3 presents study measures and response distributions regarding dilemmas and experiences with prognosis-related communication. In regard to ethical dilemmas, the most frequently reported items included participants reporting that they always or often felt pressure not to provide information due to a concern of contradicting what doctors said (180/373, 48.3%) and that social custom and cultural barriers prevent them from sharing prognostic information (167/373, 44.8%). Oncology nurses in our study reported that they experienced a relatively moderate level of ethical dilemmas in prognosis communication (mean 13.5, SD 3.43; range 5‐20). For prognosis-related communication experiences, 71% (265/373) of the participants reported that families always or often requested withholding communication from patients, and 53.4% (199/373) indicated that nurses were always or often not encouraged to participate in prognosis communication. The mean score of prognosis-related communication experiences was 10.64 (SD 2.49).

**Table 3.** Study measures (dilemmas and experiences with prognosis-related communication) and response distributions (n=373).

Items	Never/almost never	Rarely	Sometimes	Often	Always/almost always
Dilemmas in prognosis-related communication, n (%)					
Feel pressure to not provide information about prognosis to patients to avoid contradicting what the doctors have said	16 (4.3)	49 (13.1)	128 (34.3)	110 (29.5)	70 (18.8)
Avoid talking with patients about prognosis-related information due to the discomfort in giving bad news	19 (5.1)	49 (13.1)	149 (39.9)	95 (25.5)	61 (16.4)
Social customs/cultural barriers prevent you from sharing prognosis-related information	17 (4.6)	50 (13.4)	139 (37.3)	105 (28.2)	62 (16.6)
Ethically conflicted when patients or family ask about prognosis-related communication	19 (5.1)	59 (15.8)	148 (39.7)	85 (22.8)	62 (16.6)
Prognosis-related communication experiences, n (%)					
Patients do not want their family members to be told of their prognosis	22 (5.9)	72 (19.3)	118 (31.6)	106 (28.4)	55 (14.7)
Families/relatives request that the patient is not told bad news	8 (2.1)	26 (7.0)	74 (19.8)	171 (45.8)	94 (25.2)
Nurses are not encouraged to be involved in breaking bad news in my area	27 (7.2)	57 (15.3)	90 (24.1)	130 (34.9)	69 (18.5)

Table 4 presents study measures and response distributions regarding attitudes toward and barriers to prognosis-related communication. In terms of attitudes, the majority of participants were positive toward prognosis-related communication. For example, the majority agreed or strongly agreed that oncology nurses were responsible for helping patients prepare for their EOL care (330/373, 88.5%) and that prognosis communication can help patients make a timely decision about their treatments (328/373, 87.9%). The mean score for attitudes toward prognosis-related communication was 18.84 (SD 3.65). In regard to barriers for prognosis-related communication, the items that the participants mostly agreed or strongly agreed with included worries about taking away patients’ hope (317/373, 85%), followed by feeling uncertain about their roles (309/373, 82.8%). The overall mean barrier score was 12.0 (SD 2.50).

**Table 4.** Study measures (attitudes toward and barriers to prognosis-related communication) and response distributions (n=373).

Items	Strongly disagree	Disagree	Agree	Strongly agree
Attitudes toward prognosis-related communication, n (%)				
Patients can make timely decisions about their treatments if they understand their prognosis.	10 (2.7)	35 (9.4)	158 (42.4)	170 (45.6)
Patients can make timely decisions about hospice enrollment if they understand their prognosis.	24 (6.4)	24 (6.4)	159 (42.6)	166 (44.5)
I feel it is my responsibility to initiate a discussion with physicians about a patient’s prognosis if the patient has questions about his or her prognosis.	13 (3.5)	43 (11.5)	213 (57.1)	104 (27.9)
I feel that oncology nurses have a responsibility to help patients prepare for their end of life.	21 (5.6)	22 (5.9)	154 (41.3)	176 (47.2)
I am willing to initiate a discussion with patients regarding prognosis-related information.	22 (5.9)	57 (15.3)	193 (51.7)	101 (27.1)
I feel that answering questions about prognosis-related information is within the scope of nursing practice.	25 (6.6)	77 (20.6)	187 (50.1)	84 (22.5)
Barriers to prognosis-related communication, n (%)				
Uncertainty about my role in communicating prognosis-related information is a major barrier to helping patients and families understand their prognosis	22 (5.9)	42 (11.3)	219 (58.7)	90 (24.1)
Lack of time is a major barrier to discussing prognosis-related information with patients and families.	17 (4.6)	72 (19.3)	173 (46.4)	111 (29.8)
Fear of taking away patients’ hope is a major barrier to discussing prognosis-related information with patients and families.	15 (4)	41 (11)	199 (53.4)	118 (31.6)
Physician discomfort with giving bad news is a major barrier to helping patients and families understand their prognosis.	18 (4.8)	102 (27.3)	174 (46.6)	79 (21.2)

### Factors Impacting Ethical Dilemmas in Prognosis-Related Communication

Table 5 summarizes the results of the ordinary least squares regression analysis. Previous experience with prognosis communication (*β*=0.46; *P*<.001), perceived barriers (*β*=0.40; *P*<.001), and years of work experience (*β*=–0.14; *P*=.002) were significant predictors of experiencing ethical dilemmas when engaging in prognosis-related communication. These findings suggest that participants who had more negative experiences or perceived more barriers to prognosis communication encountered more dilemmas. Participants with fewer years of experience as oncology nurses were more likely to experience a dilemma. All other variables, including attitudes, formal training, and demographic covariates did not significantly predict dilemmas in prognosis-related communication. This model explained a substantial portion of the variance in ethical dilemmas in prognosis communication (*R*=0.79; *R*^2^=0.62; adjusted *R*^2^=0.61; *F*_9,353_=63.13; *P*<.001).

**Table 5.** Predictors for dilemmas in prognosis-related communication (N=373).

Predictor	*b^a^*	SE	β^b^ (95% CI)		*P* value
Barriers	0.55	0.07	0.40 (0.41-0.69)		<.001
Experiences with prognosis communication	0.62	0.06	0.46 (0.50-0.73)		<.001
Attitudes	–0.02	0.05	–0.02 (–0.12 to 0.07)		.66
Marital status	–0.10	0.07	–0.05 (–0.25 to 0.05)		.18
Age	0.04	0.03	0.05 (–0.03 to 0.10)		.23
Sex	–0.80	0.50	–0.05 (–1.80 to 0.19)		.11
Level of education	–0.27	0.17	–0.06 (–0.60 to 0.06)		.10
Years of working as an oncology nurse	–0.11	0.03	–0.14 (–0.18 to –0.04)		.002
Formal training	–0.20	0.13	–0.05 (–0.45 to 0.06)		.13

a Regression coefficient.

b Standardized regression coefficient.

## Discussion

### Prognosis-Related Practice

Our study suggests that prognosis disclosure is not a simple dichotomy of telling versus not telling, but rather it varies by the degree of information provided. In this study, about 89% (332/373) of the participants reported either fully or partially engaging in prognosis communication with patients or their families, which is higher than a previous study in Taiwan where about 71% of nurses reported doing so [27], but it is lower than another study in China where 97.2% of oncology nurses engaged in truth-telling [28]. However, consistent with a previous study [8], our study revealed that full disclosure of a prognosis was more frequently given to families (163/373, 43.7%) rather than to patients (75/373, 20.1%). Disclosure of a patient’s prognosis to the family before the patient may be intended to protect the patient from the potential harm associated with receiving negative news [29]. Although the frequency of full prognosis disclosure to families was twice the frequency of disclosure to patients, only 43.7% (163/373) of participants fully informed the family about the patient’s prognosis. Further analysis revealed that 21.5% (80/373) of the participants provided only partial information to both patients and family members, while 11% (41/373) avoided engaging in prognostic discussions with either group. This hesitation may stem from various factors, including personal discomfort, lack of training, and institutional policies or protocols that affect information delivery [30]. Future studies exploring the reasons behind the varying degrees of prognosis disclosure would be valuable.

### Ethical Dilemmas in Prognosis-Related Communication

Oncology nurses in our study experienced a relatively moderate level of ethical dilemmas in cancer prognosis-related communication. Ethical dilemmas in health care arise from various factors, including the influence of sociocultural values and traditions on professional practice, as well as personal attitudes [12,31]. In China, it is traditional for physicians to take primary responsibility for informing patients and their families of the diagnosis and prognosis, which may cause nurses to hesitate when it comes to challenging authority or stepping beyond their professional roles. Nurses may also fear providing inaccurate information without a complete understanding of the patient’s prognosis. Furthermore, sociocultural values rooted in Confucianism, which emphasize protecting patients, maintaining family harmony, and prioritizing family-centeredness, can create conflicts for health care professionals in promoting patient-informed decision-making [16,17]. This may lead to situations where patient autonomy is compromised to honor cultural and social traditions and expectations.

### Predictors of Ethical Dilemmas in Prognosis-Related Communication

One significant predictor for ethical dilemmas about prognostic communication was the perceived barriers to communication. Nurses who perceived a greater level of barriers were more likely to experience dilemmas. This finding aligns with previous studies indicating that health care professionals’ discomfort and the burden of breaking bad news stem from their concerns about patients’ inability to cope and relational distress [3]. Our study participants’ uncertainty about their role might stem from the hierarchy in the health care system in China, where physicians are traditionally expected to lead discussions about prognosis [15,27]. Nevertheless, conflicting informed consent laws and regulations in China, which emphasize a patient’s right to know but discourage health care professionals from truth-telling if it could cause adverse events, create fear of lawsuits and conflict with the family [16]. Consequently, the discomfort and lack of engagement among health care professionals in communicating a prognosis may impose additional burdens and challenges on nurses when patients or family members seek information.

Another significant predictor of dilemmas surrounding prognosis-related communication was the nurses’ experience with prognosis communication. Negative experiences, such as families requesting that the prognosis be withheld from patients, were positively associated with experiencing dilemmas. Previous studies also supported that family requests to withhold prognosis information hinder health care professionals’ disclosure of this information to patients [8,14,20]. However, deciding whether to disclose a prognosis is complex, requiring a balance between the patient’s wishes and family’s concerns. Family members’ preferences might not align with the patient’s preferences, creating conflicts with health care professionals. Health care professionals’ assessment of both patients’ and families’ prognostic information preference is important to reduce conflicts associated with their discordant views [32]. Regardless, family is an important source of support and patients value positive relationships with their family members. Hence, integrating family into palliative and EOL care is important [14].

In addition, engaging the entire health care team in the communication process and decision-making serves as a collective approach to address ethical dilemmas [10]. Physicians are primarily tasked with delivering unfavorable news yet often evade this duty due to personal discomfort, concerns regarding patients’ psychological well-being, time constraints, and inadequate communication training [17,28]. This perhaps compels nurses to feel responsible for filling the informational voids. A recent study with oncology nurses in China highlighted the benefits of an interdisciplinary approach, especially collaborations between physicians and nurses, such as sharing patient information [28]. An interprofessional approach to prognosis communication can effectively empower nurses to communicate truthfully and foster enhanced collaboration with physicians.

The number of years of employment as an oncology nurse was another significant factor associated with experiencing dilemmas surrounding prognosis-related communication. Previous studies have shown that years of nursing experience has a significant association with communication [33] and confidence in palliative and EOL care [34]. Oncology nurses with limited experience encountered uncertainty when addressing prognosis-related inquiries, which engendered apprehension about inadvertently imparting inaccurate information to the patients [35]. Conversely, oncology nurses with extensive experience are likely to have developed advanced communication skills, attributed to their heightened exposure to patients with advanced cancer. Therefore, involving experienced nurses in communication training might be beneficial. Previous studies with Chinese nurses indicated a lack of education in death, dying, and palliative and EOL care [12,30]. Although formal communication training increases health care professionals’ engagement in disclosing a diagnosis and prognosis [36], it was not statistically significant in our study. This may be due to the lack of an established formal curriculum on prognosis communication, particularly culturally tailored and adapted skill training in which health care professionals, regardless of receiving formal training, may still be unsure about how to facilitate such challenging communications. In addition, the relatively young age of the study participants suggests that they may switch to other types of nursing units over the course of their careers. Therefore, providing recurring training opportunities led by experienced nurses could help improve communication skills and better equip them to address dilemmas related to challenging topics like prognosis communication.

Nurse communication training is still relatively new, and few training programs provide comprehensive skill training for palliative care. One example is the COMFORT (Connect, Options, Making Meaning, Family Caregivers, Openings, Relating, Team) framework, which outlines communication pathways for palliative care [37,38]. COMFORT incorporates communication theory into clinical research, providing a solid framework for palliative nursing communication with patients and families. Its “train-the-trainer” model has been found to improve nurses’ attitudes, comfort levels, perceived self-efficacy, and confidence in engaging in challenging topics with family caregivers [37]. Despite its promising approach, it is crucial to culturally tailor its components for successful adoption. Health care organizations should adapt the curriculum to meet the unique needs and expectations of oncology nurses while accounting for Chinese cultural nuances.

### Limitations and Future Studies

This study has several limitations that need to be acknowledged. This study included only 4 hospitals in Wuhan, China, which may limit the generalizability of the findings to oncology nurses in other provinces. Different provinces may have varying regulations and protocols regarding prognosis-related communication. In addition, the 4 hospitals where participants were recruited were tertiary hospitals, which may lead to different experiences for nurses compared to those working in primary or secondary hospitals or smaller regional hospitals. Future studies expanding study sites to include hospitals of different levels can broaden our understanding of this topic. Additionally, the psychometric properties of the measures used in this study were not empirically confirmed. Hence, future research is needed to develop reliable and valid measures.

### Conclusion

Communicating a cancer prognosis is a complex process, and ethical dilemmas that nurses encounter need to be understood within their social and cultural contexts. Strategies to address ethical dilemmas require ongoing training and interdisciplinary collaboration. Communication training tailored to specific cultural contexts is indispensable within health care settings. Without uniform or unified policies, gaps and dilemmas in practice inevitably arise, potentially compromising patient care. Given the diverse preferences of patients and their families, communication about prognosis must be individualized and sensitive to their unique needs and backgrounds. Effective communication about cancer prognosis requires a collaborative effort centered around the patient. By harnessing their expertise and using tools that guide the understanding of patient preferences, health care professionals can ensure that discussions are informative, respectful, and supportive. Ultimately, emphasizing teamwork and ethical awareness enhances the quality of prognosis discussions and promotes the well-being of both patients and providers.

## Abbreviations

COMFORT: Connect, Options, Making meaning, Family caregivers, Openings, Relating, Team
EOL: end-of-life
